# ^18^F-flortaucipir (AV-1451) tau PET in frontotemporal dementia syndromes

**DOI:** 10.1186/s13195-019-0470-7

**Published:** 2019-01-31

**Authors:** Richard M. Tsai, Alexandre Bejanin, Orit Lesman-Segev, Renaud LaJoie, Adrienne Visani, Viktoriya Bourakova, James P. O’Neil, Mustafa Janabi, Suzanne Baker, Suzee E. Lee, David C. Perry, Lynn Bajorek, Anna Karydas, Salvatore Spina, Lea T. Grinberg, William W. Seeley, Eliana M. Ramos, Giovanni Coppola, Maria Luisa Gorno-Tempini, Bruce L. Miller, Howard J. Rosen, William Jagust, Adam L. Boxer, Gil D. Rabinovici

**Affiliations:** 1 Memory and Aging Center, University of California at San Francisco, 675 Nelson Rising Lane, Suite 190, San Francisco, CA USA; 2 Helen Wills Neuroscience Institute, University of California at Berkeley, Berkeley, USA; 3 Life Sciences Division, Lawrence Berkeley National Laboratory, Berkeley, USA; 4 Departments of Psychiatry and Neurology, Semel Institute for Neuroscience and Human Behavior, David Geffen School of Medicine, University of California, Los Angeles, CA USA

**Keywords:** Biomarkers, Frontotemporal dementia, Tau imaging, Neuropathology, Tau

## Abstract

**Background:**

The tau positron emission tomography (PET) ligand ^18^F-flortaucipir binds to paired helical filaments of tau in aging and Alzheimer’s disease (AD), but its utility in detecting tau aggregates in frontotemporal dementia (FTD) is uncertain.

**Methods:**

We performed ^18^F-flortaucipir imaging in patients with the FTD syndromes (*n* = 45): nonfluent variant primary progressive aphasia (nfvPPA) (*n* = 11), corticobasal syndrome (CBS) (*n* = 10), behavioral variant frontotemporal dementia (bvFTD) (*n* = 10), semantic variant primary progressive aphasia (svPPA) (*n* = 2) and FTD associated pathogenic genetic mutations microtubule-associated protein tau (*MAPT*) (*n* = 6), chromosome 9 open reading frame 72 (*C9ORF72*) (*n* = 5), and progranulin (*GRN*) (*n* = 1). All patients underwent MRI and β-amyloid biomarker testing via ^11^C-PiB or cerebrospinal fluid. ^18^F-flortaucipir uptake in patients was compared to 53 β-amyloid negative normal controls using voxelwise and pre-specified region of interest approaches.

**Results:**

On qualitative assessment, patients with nfvPPA showed elevated ^18^F-flortacupir binding in the left greater than right inferior frontal gyrus. Patients with CBS showed elevated binding in frontal white matter, with higher cortical gray matter uptake in a subset of β-amyloid-positive patients. Five of ten patients with sporadic bvFTD demonstrated increased frontotemporal binding. *MAPT* mutation carriers had elevated ^18^F-flortaucipir retention primarily, but not exclusively, in mutations with Alzheimer’s-like neurofibrillary tangles. However, tracer retention was also seen in patients with svPPA, and the mutations *C9ORF72*, *GRN* predicted to have TDP-43 pathology. Quantitative region-of-interest differences between patients and controls were seen only in inferior frontal gyrus in nfvPPA and left insula and bilateral temporal poles in *MAPT* carriers. No significant regional differences were found in CBS or sporadic bvFTD. Two patients underwent postmortem neuropathological examination. A patient with *C9ORF72*, TDP-43-type B pathology, and incidental co-pathology of scattered neurofibrillary tangles in the middle frontal, inferior temporal gyrus showed corresponding mild ^18^F-flortaucipir retention without additional uptake matching the widespread TDP-43 type B pathology. A patient with sporadic bvFTD demonstrated punctate inferior temporal and hippocampus tracer retention, corresponding to the area of severe argyrophilic grain disease pathology.

**Conclusions:**

^18^F-flortaucipir in patients with FTD and predicted tauopathy or TDP-43 pathology demonstrated limited sensitivity and specificity. Further postmortem pathological confirmation and development of FTD tau-specific ligands are needed.

**Electronic supplementary material:**

The online version of this article (10.1186/s13195-019-0470-7) contains supplementary material, which is available to authorized users.

## Background

Pathologic tau with three or four repeat (3R and 4R) microtubule binding domains aggregates intracellularly into paired helical filaments (PHF) in Alzheimer’s disease and twisted ribbons or straight filaments in a range of frontotemporal dementia (FTD) syndromes [1]. Tau aggregates are the underlying pathology in the majority of patients presenting clinically with progressive supranuclear palsy (PSP), corticobasal syndrome (CBS) and nonfluent variant primary progressive aphasia (nfvPPA), and up to one third of patients presenting with behavioral variant frontotemporal dementia (bvFTD) [2–5]. The underlying pathological process of frontotemporal lobar degeneration (FTLD) can be associated with tau aggregates in Pick’s disease, corticobasal degeneration (CBD), PSP, argyrophilic grain disease (AGD), and globular glial tauopathy (GGT) [6]. Conversely, TAR DNA-binding protein (TDP-43) pathology is seen in the majority of patients with semantic variant primary progressive aphasia (svPPA) and frontotemporal dementia with amyotrophic lateral sclerosis (FTD-ALS) [3, 7]. Familial FTD can be caused by mutations in the microtubule-associated protein tau gene (*MAPT*) with resulting tau pathology, or by mutations in progranulin (*GRN*) gene or chromosome 9 open reading frame 72 gene (*C9ORF72*), resulting in TDP-43 pathology [8–10].

Recent clinical trials for neurodegenerative disease have focused on reducing pathological protein aggregates [11], with novel therapies entering clinical trials in Alzheimer’s disease and PSP. Analogous to the instrumental role of β-amyloid PET in anti-amyloid therapeutic trials [12], an imaging marker that can detect and quantify tau could advance the development of anti-tau therapies by enabling appropriate subject selection, early intervention, and assessment of target engagement.

The PET tracer ^18^F-flortaucipir (previously ^18^F-T807 and ^18^F-AV1451) binds in-vitro with high affinity to neurofibrillary tangles (NFT) in Alzheimer’s disease composed of 3R/4R PHF [13–15]. In vivo, ^18^F-flortaucipir retention in Alzheimer’s disease matches the expected distribution of tau pathology in Alzheimer’s disease, correlating with clinical symptoms and neurodegeneration [16–18]. In FTD and related tauopathies, in vivo findings with ^18^F-flortaucipir have been mixed, with some suggesting ^18^F-flortaucipir retention in areas of predicted pathology in CBS and *MAPT* mutation carriers [19–23]. In PSP, most studies demonstrate ^18^F-flortaucipir retention correlating with areas of predicted tau neuropathology, differentiating PSP patients from normal subjects and patients with Alzheimer’s or Parkinson’s disease at a group level, though negative results have also been reported [24–28]. Of note, ^18^F-flortaucipir retention has been demonstrated in patients with svPPA, raising concerns for non-tau binding [29, 30]. In-vitro ^18^F-flortaucipir binding studies non-Alzheimer’s tauopathies have yielded conflicting results, with some suggesting no autoradiography binding on postmortem FTD tauopathy tissue, even in areas with in vivo image uptake [15, 31], while others propose present but weak binding to some tau aggregates [14, 22, 32]. The interpretation of in vivo retention in brain areas relevant to FTD tauopathies is further complicated by “off-target” binding seen in normal controls in midbrain and basal ganglia, possibly reflecting a proclivity to bind to neuromelanin containing cells or mineralized tissue [14, 33, 34].

Building on previous reports that have focused on single syndromes, we report our center’s experience with ^18^F-flortaucipir in 45 patients representing the clinical spectrum of FTD, *MAPT, GRN*, and *C9ORF7*2 mutations. We sought to characterize the distribution, frequency, and intensity of ^18^F-flortaucipir uptake and then compare the results to a group of cognitively normal individuals and to the expected distribution of tau pathology in each syndrome. We also compare PET binding to autopsy findings in two patients: one with sporadic bvFTD and another with bvFTD caused by *C9ORF72* expansion.

## Methods

### Participants

Consecutive patients were recruited from FTD research cohorts followed at the University of California San Francisco (UCSF) between September 2014 and August 2017. All patients received a neurological history, physical, caregiver interview, neuropsychology assessment, and MRI. Diagnosis was made by consensus panel, utilizing the latest diagnostic criteria for bvFTD [35], primary progressive aphasia [36], and CBS [37]. Our center’s experience with ^18^F-flortaucipir in PSP was previously reported as part of a multi-site study [28]. One *MAPT* V337 M mutation carrier in the present series was in a previous report [23]. Clinical diagnosis incorporated MRI findings, as required in diagnostic criteria, but was blinded to PET results. Patients also had β-amyloid status assessed either via ^11^C-PiB PET (*n* = 42) or cerebrospinal fluid Aβ_42_ levels (*n* = 1) using previously described methods [38].

Normal controls were recruited from the Berkeley Aging Cohort. The eligibility criteria for controls include normal performance on cognitive tests, absence of neurological, psychiatric illnesses and lack of major illnesses, and medications that affect cognition. We selected controls that were β-amyloid negative by ^11^C-PiB. Individuals below age 60 were not scanned with ^11^C-PiB due to low likelihood of β-amyloid positivity and to minimize radiation exposure. Informed consent was obtained from all subjects or their surrogate decision-makers, and the UCSF, University of California Berkeley (UCB) and Lawrence Berkeley National Laboratory (LBNL) Institutional Review Boards for human research approved the study.

All subjects underwent MRI and PET imaging with the ^18^F-flortaucipir tracer. The mean (standard deviation) time difference between PET and MRI imaging and clinical evaluation was 1 (± 1.5) and 1 (± 1.6) month respectively.

### Genomic testing

Genomic DNA was extracted blood using standard protocols (Gentra Pure-Gene Blood Kit, QIAGEN, Inc., Valencia, CA, USA). Targeted coding (exon) and flanking noncoding region (including intron-exon boundaries, 3′ and 5′ UTR sequencing of *MAPT*) as part of a panel of ~ 300 genes implicated in neurodegenerative disease was performed. Sequencing was performed using a custom-designed Nimblegen SeqCap EZ Choice (Roche) library and sequenced on an Illumina HiSeq2500 at the University of California Los Angeles Neuroscience Genomics Core to > 70× coverage. Sequencing data was processed using the Broad Institute’s Genomic Analysis Toolkit (GATK) best practices pipeline [39].

### MRI

All normal control subjects underwent high-resolution T1-weighted magnetization prepared rapid gradient echo (MPRAGE) scan on a 1.5-T Siemens Magnetom Avanto scanner at LBNL (slice thickness = 1.0 mm with 50% gap; in-plane resolution = 1.0 × 1.0 mm; matrix = 256 × 256; repetition time = 2110 ms; echo time = 3.58 ms; inversion time = 1100 ms; flip angle = 15°). Patients underwent T1-weighted MPRAGE sequence on a 3-T Siemens Tim Trio/Prisma scanner at the UCSF Neuroimaging Center (slice thickness = 1.0 mm; in-plane resolution = 1.0 × 1.0 mm; matrix = 240 × 256; repetition time = 2300 ms; echo time = 2.98 ms; inversion time = 900 ms; flip angle = 9°). T1-MRI images were first segmented, parcellated with FreeSurfer 5.3 (http://surfer.nmr.mgh.harvard.edu/), and spatially normalized to the Montreal Neurological Institute (MNI) space using Statistical Parametric Mapping Version 12 (SPM12) (http://www.fil.ion.ucl.ac.uk/spm/software/spm12/;Wellcome Department of Imaging Neuroscience, Institute of Neurology, London, England).

### Positron emission tomography

PET scans were performed at LBNL on a Siemens Biograph Truepoint PET/CT scanner in 3D acquisition mode with a low-dose CT scan performed prior for attenuation correction. PET images were reconstructed using an ordered subset expectation maximization algorithm with weighted attenuation scatter correction and smoothed with a 4-mm Gaussian kernel (calculated image resolution 6.5 × 6.5 × 7.25 mm using Hoffman) [17]. Radiotracers were synthesized and radiolabeled at the LBNL Biomedical Isotope Facility as previously described [40]. For ^18^F-flortaucipir, 10 mCi of tracer was injected and data from 80 to 100 min post injection was used. For ^11^C-PiB, 15 mCi of tracer was injected and dynamic acquisition 0 to 90 min post injection was acquired for all normal controls and most patients. Due to procedure intolerance, some patients underwent 50–70 min acquisition and standardized uptake value ratio (SUVR) was calculated instead. Both ^18^F-flortaucipir and ^11^C-PiB images were evaluated prior to analysis for motion and adequacy of statistical counts.

### Image processing

Neuroimaging data processing was performed using SPM12, implemented in MATLAB 8.3 (MathWorks, Sherborn, MA) and on FreeSurfer 5.3.

For ^11^C-PiB, distributed volume ratio (DVR) was calculated using Logan graphical analysis with the gray matter cerebellum time-activity curve used as a reference tissue input function while SUVR was calculated by dividing the mean 50–70 min post injection uptake by the gray matter cerebellar mask [41]. A DVR value above 1.07 was determined to be positive for normal controls [42]. For patients with FTD syndromes, positivity for Aβ was determined both by visual read via an experienced neurologist (GDR) and a DVR > 1.07 or SUVR > 1.21.

^18^F-flortaucipir PET frames were realigned and co-registered onto their corresponding native space MRIs. SUVR maps were calculated using inferior cerebellar gray as reference region, created by defining the overlap between FreeSurfer’s cerebellar gray parcel and inferior cerebellum parcels from the SUIT atlas [43–46]. For voxelwise comparisons, PET SUVR images were spatially normalized to MNI space using the deformation parameters defined using the corresponding MRI and smoothed by a Gaussian kernel of 4 mm full width at half maximum (FWHM). PET images were masked before smoothing to exclude non-gray, non-white matter voxels, limiting the introduction of skull signal into gray and white matter tissue.

All ^18^F-flortaucipir SUVR images were reviewed qualitatively by two neurologists (RMT and GDR) and a radiologist (OSL) experienced in neurodegenerative syndromes and PET imaging, to assess for areas of elevated binding. Positive scans were determined via visual assessment of the level of tracer binding vis-à-vis normal controls and anatomic prediction of pathological protein aggregates. Specifically, confluent PET signal in cortical gray or subcortical white matter was considered abnormal. Patterns of tracer retention that were expected a priori included asymmetric peri-Sylvian and inferior frontal uptake in nfvPPA, frontal or anterior temporal uptake in sporadic and genetic bvFTD, and frontal and parietal gray and white matter in CBS. Visual raters did not categorize images as abnormal solely based on basal ganglia, thalamus, or midbrain uptake, as these regions are known to show “off-target” binding also seen in normal controls [14, 33, 34]. Clinicians were not blinded to clinical information when reviewing images. Qualitative descriptions described below represent consensus conclusions.

### Autopsy examination

Postmortem brains were processed and diagnoses were formulated according to neuropathological criteria previously described [47–50]. Fixed tissue slabs were dissected into blocks representing dementia-relevant brain regions, embedded in paraffin wax and cut into 8-μm-thick sections. Regional neurodegeneration as defined by mircovacuolation, astrogliosis, neuronal loss was assessed using hematoxylin and eosin-stained sections, while proteinopathies were stained with antibodies for hyperphosphorylated tau (CP13, 1:1000 mouse monoclonal, from Dr. Peter Davies), 3R tau (3R, antimouse, 1:500, Millipore, Billerica, MA), β-amyloid (anti-amyloid β, antimouse, 1:150, Millipore), TDP-43 (antirabbit, 1:4000, Proteintech Group, Chicago, IL), and α-synuclein (antimouse, 1:1000, Millipore). Pathological assessment was performed blinded to ^18^F-flortaucipir results.

### Statistics

#### Voxelwise contrast between patients and controls

Group comparisons were performed in SPM12 to assess voxelwise differences in ^18^F-flortaucipir SUVR between normal controls and patients with (i) nfvPPA and (ii) CBS. These two syndromes were chosen due to larger sample size availability, relative homogeneity of PET images, and high likelihood of underlying tauopathy. CBS images were flipped so that sides contralateral (CL) to symptom onset were aligned. Age was included as a covariate in the statistical model and a threshold of *p* < 0.001 uncorrected together with a cluster extent of 200 voxels (675 mm^3^) or family-wise error multiple comparison correction pFWE < 0.05 was used.

#### Region of interest analyses

We defined a priori regions of interest (ROI) for each clinical syndrome for group comparisons between ^18^F-flortaucipir SUVR values in patients versus controls. Regions were defined in native space using the FreeSurfer Desikan Atlas [43]. Syndrome-specific ROIs were defined for each syndrome based on areas of most severe neurodegeneration in the literature, while avoiding areas of “off-target” binding in the basal ganglia, midbrain, and bilateral regions were analyzed separately. Given the potential for overlapping phenotypes in FTLD, all selected gray matter ROIs were also applied to the remaining FTD syndromes in an exploratory ROI analysis. The selected ROI for each syndrome were:

i. nfvPPA: pars opercularis, pars triangularis, precentral and superior frontal gyrus [51],
ii. CBS: precentral gyrus, rostral and caudal middle frontal gyrus. We also included the white matter voxels underlying the caudal middle frontal gyrus gray matter using white matter labeling by proximity to cortical folds [52, 53],
iii. *MAPT* carriers and patients with bvFTD: insula, and meta-ROIs (created from Desikan Atlas regions) in orbitofrontal (medial, lateral orbitofrontal regions) and temporal cortex (all temporal regions) [4, 54–56],
iv. *C9ORF72* and *GRN* carriers: orbitofrontal cortex, insula and precentral gyrus [54, 57],
v. svPPA: temporal poles, insula and orbitofrontal cortex [58].

In addition, to illustrate the ^18^F-flortaucipir SUVR differences between Alzheimer’s disease and FTD, temporal cortex and precentral gyrus SUVR from a cohort of age, sex, and disease severity matched Alzheimer’s disease subjects was compared to all FTD subjects and normal controls.

#### *W*-score and *w*-score frequency map generation

As nonspecific increase in ^18^F-flortaucipir retention can be seen with age in normal controls, we generated *w*-scores to quantify the degree of ^18^F-flortaucipir abnormality in each voxel A group of 53 healthy controls (described above) was chosen as a reference to transform patients’ SUVR maps into *w*-score maps [59–61]. A *w*-score is a modified *z*-score adjusted for covariates of interest (in this case, age) and was computed in two steps [62, 63]. First, a voxelwise regression model was derived from the control group to estimate the effect of age on each voxel’s SUVR. Individual *w*-maps were then computed by subtracting each voxel’s raw SUVR from regression model predicted SUVR, then divided by a map representing the standard deviation of the regression model residuals. The resulting *w*-map therefore indicates the value of each voxel in relation to normal controls of the same age. We used a threshold of 1.65, corresponding to the 95th percentile of normal distribution, as the threshold to designate abnormal voxels.

*W*-score frequency maps were analyzed at the group level by generating frequency maps to examine the proportion of patients in each clinical group who had abnormally elevated binding for their age. Each patient’s *w*-map was binarized at a threshold of 1.65 such that voxels above the threshold were assigned a value of 1. The binarized *w*-score frequency maps were then summed for all patients in a specific clinical diagnosis to generate the frequency maps, which illustrate the prevalence of positive ^18^F-flortaucipir regional retention in a given FTD syndrome.

#### Statistical analyses

Differences in baseline characteristics were assessed using Fisher’s Exact test for categorical data, Kruskal-Wallis test and post hoc Dunn’s pairwise comparison with Bonferroni test adjustment for nonparametric data. Differences in ROI ^18^F-flortaucipir uptake for each diagnosis were compared to normal controls using Mann-Whitney *U* test. To assess whether differences in hemispheric SUVR corresponded to symptom laterality, an asymmetric index (AI) was also calculated for patients with CBS in the precentral gyrus using the formula AI = 200 × (IL uptake – CL uptake)/(IL uptake + CL uptake), where IL is the side ipsilateral to symptom onset and negative values indicate increased CL hemispheric uptake compared to IL. To designate abnormal asymmetry, we adopted a threshold index of 1.91, corresponding to the maximum asymmetry index seen in our normal controls.

## Results

### Patients

Eleven nfvPPA, 10 CBS, six *MAPT* carriers, 10 sporadic bvFTD, five *C9ORF72* carriers, two svPPA, and one *GRN* carrier were included. One patient with nfvPPA was excluded due to poor PET image quality. Fifty-three individuals between age 20 and 93 years old were included in the control group, based on normal cognitive assessment for every individual and a negative ^11^C-PiB PET for those above 60 years old. Demographic and clinical characteristics are presented in Table 1. As expected, normal controls had higher MMSE than patients with bvFTD, nfvPPA, and *C9ORF72*, *GRN*, *MAPT* carriers, while patients with nfvPPA had lower Clinical Dementia Rating scale Sum of Boxes (CDR-SB) scores compared to patients with bvFTD and *MAPT* carriers. One patient with CBS received neuroimaging studies but declined further testing. ^11^C-PiB SUVR was used to determine β-amyloid positivity for 14 patients instead of DVR. ^11^C-PiB imaging for one *C9ORF72* carrier, one patient with sporadic bvFTD and one *MAPT* carrier were not available. β-amyloid status for the *MAPT* carrier was determined via CSF Aβ_42_ level.

**Table 1:** Subject demographics

	nfvPPA	CBS	MAPT	bvFTD	C9ORF72, GRN	svPPA	All patients	NC
*N*	11	10	6	10	6	2	45	53
Age	64 (56, 75)	68 (54, 77)	52 (37, 68)	68 (34, 78)	61 (48, 71)	59, 71	63 (34, 78)	76 (20, 93)
Education	15 (12, 24)	16 (13, 20)^┼^	16 (14, 18)	16 (12, 20)	18 (12, 20)	12, 18	16 (12, 24)	17 (13, 20) ^┼^
Handedness (R/L)	8/3	9/1	6/0	9/1	6/0	2/0	40/5	49/3^┼^
Sex (F/M)	8/3	5/5	2/4	1/9	4/2	1/1	21/24	23/30
MMSE	26 (17, 30)	27 (9, 30) ^┼^	18 (4, 30)	22 (18, 29)	26 (21, 26)	25, 28	24 (4, 30)	29 (25, 30)*
CDR-SB	0.8 (0, 3)**	2 (1, 7) ^┼^	7 (1, 10)	7 (1.5, 12)	6 (0.5, 8)	3.5, 3.5	3.9 (0, 12)	NA
Aβ (+/−)	0/11	4/6	1/5	3/6^┼^	1/4^┼^	0/2	9/34	0/44^‡^
ApoE E4 (+/−)	2/9	4/5^┼^	2/3^┼^	0/10	2/4	0/2	10/33	7/43^┼^

*nfvPPA* nonfluent variant primary progressive aphasia, *CBS* corticobasal syndrome, *MAPT* microtubule associated protein tau, *bvFTD* behavioral variant frontotemporal dementia, *C9ORF72* chromosome 9 open reading frame 72 gene, *GRN* progranulin, *svPPA* semantic variant primary progressive aphasia, *NC* normal control, *MMSE* Mini-Mental State Examination, *CDR-SB* clinical dementia rating score sum of boxes, *NA* not applicable, *Aβ* β-amyloid status by Pittsburgh compound B PET scan or CSF Aβ_42_ < 250 pg/ml

Median (min, max)

^┼^Missing data

*NC higher than bvFTD, nfvPPA, MAPT, C9ORF72 and PGRN and carriers *p* < 0.05

**nfvPPA lower than bvFTD and MAPT carriers *p* < 0.05

^‡^Aβ status not available in 9 subjects due to age < 60

### Nonfluent variant primary progressive aphasia

**Fig. 1:**
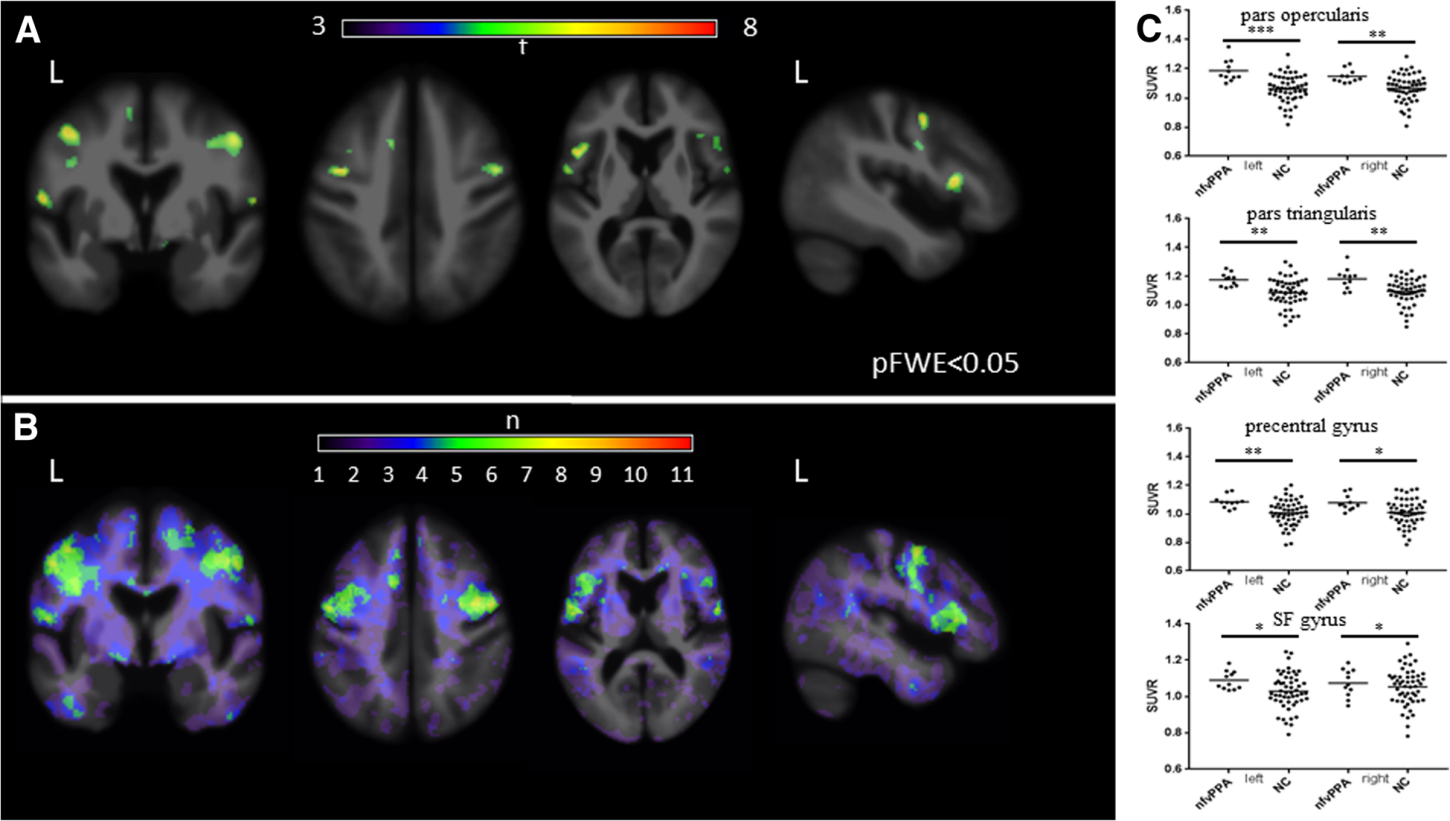
^18^F-flortaucipir in nfvPPA. **a** Voxel-wise contrast (pFWE< 0.05) of 11 nfvPPA patients with 53 normal controls with age as covariate. **b** *W*-score frequency map showing number of patients with suprathreshold age-adjusted SUVR values (*w*-score ≥ 1.65) compared to normal controls. Peak voxels suggest eight of 11 subjects have elevated *w*-score voxels in the same region. **c** ^18^F-flortaucipir SUVR values for each pre-specified region of interest; horizontal bar denotes mean. L, left; NC, normal control; SF, superior frontal. ***p ≤ 0.001, **p ≤ 0.01, *p ≤ 0.05

Representative ^18^F-flortaucipir images from 11 patients with nfvPPA and the corresponding single-subject *w*-score maps are shown in Additional file 1: Figure S1. Tracer retention in the frontal operculum, inferior or middle frontal gyrus was seen in all scans to varying degrees. Patients 1–7 showed additional bilateral but asymmetric frontal white matter binding, while patients 8–11 demonstrated mild uptake in the prefrontal cortex. All scans show varying degrees of uptake in the bilateral basal ganglia. On voxelwise comparison to normal controls, nfvPPA demonstrated increased uptake in left greater than right frontal operculum, middle, inferior frontal gyri and left superior frontal gyri (pFWE< 0.05) (Fig. 1a). The *w*-score frequency map demonstrated elevated *w*-scores above 1.65 in bilateral middle frontal gyri and frontal operculum in approximately two thirds of patients scanned, with voxels above 1.65 in 8 of 11 patients in peak areas (Fig. 1b). ROI analyses revealed group differences in nfvPPA compared to normal controls in the bilateral pars opercularis (left *p* = 0.0001, right *p* = 0.0018), pars triangularis (left *p* = 0.0016, right *p* = 0.0029), precentral gyrus (left *p* = 0.003, right *p* = 0.0112), and superior frontal gyrus (left *p* = 0.03, right *p* = 0.045). Of note, while there was substantial overlap in SUVR values between patients and controls, very few patients with nfvPPA had SUVR below the normal control mean SUVR in the selected ROIs (Fig. 1c). Exploratory comparisons further revealed increased SUVR in the left caudal middle frontal gyrus (*p* = 0.01) (Table 2).

**Table 2:** ^18^F-flortaucipir standardized uptake value ratio in various regions of interest (for CBS, left is contralateral to symptom onset)

		CMF	RMF	SF	OF	IN	PO	PT	PC	TL	TP	WM CMF
Left or CL												
Controls *n* = 53	Mean ± sd	1.06 ± 0.1	1.06 ± 0.1	1.03 ± 0.1	1.16 ± 0.1	1.11 ± 0.12	1.06 ± 0.09	1.09 ± 0.1	1.01 ± 0.09	1.14 ± 0.09	1.12 ± 0.1	1.2 ± 0.16
	[min, max]	[0.81,1.3]	[0.84,1.26]	[0.79,1.25]	[0.79,1.33]	[0.78,1.37]	[0.82,1.3]	[0.86,1.3]	[0.78,1.2]	[0.88,1.28]	[0.8,1.29]	[0.82,1.6]
nfvPPA *n* = 11	Mean ± sd	*1.15 ± 0.09*	1.09 ± 0.05	*1.09 ± 0.05*	1.2 ± 0.05	1.13 ± 0.05	*1.19 ± 0.07*	*1.18 ± 0.05*	*1.08 ± 0.04*	1.15 ± 0.05	1.14 ± 0.09	
	[min, max]	*[1,1.37]*	[1.02,1.19]	*[1.-3.1.18]*	[1.1,1.26]	[1.05,1.24]	*[1.1,1.35]*	*[1.12,1.26]*	*[1.02,0.16]*	[1.08,1.24]	[0.97,1.31]	n/a
	*p* value	*0.01*	0.22	*0.0306*	0.19	0.84	*0.0001*	*0.0016*	*0.003*	0.88	0.53	
CBS *n* = 7	Mean ± sd	1.05 ± 0.09	1.02 ± 0.06	1.01 ± 0.07	1.18 ± 0.06	1.11 ± 0.08	1.1 ± 0.08	1.12 ± 0.06	1.06 ± 0.07	1.15 ± 0.04	1.08 ± 0.06	1.29 ± 0.14
	[min, max]	[0.95,1.19]	[0.93,1.08]	[0.91,1.11]	[1.09,1.16]	[1.04,1.28]	[1,1.26]	[1.05,1.2]	[0.96,1.13]	[1.07,1.2]	[0.99,1.16]	[1.41,1.56]
	*p* value	0.59	0.29	0.44	0.65	0.77	0.44	0.36	0.09	0.79	0.38	0.33
MAPT *n* = 6	Mean ± sd	1.11 ± 0.33	1.07 ± 0.12	1.02 ± 0.27	1.22 ± 0.1	*1.21 ± 0.1*	1.14 ± 0.19	1.12 ± 0.13	0.96 ± 0.2	1.21 ± 0.1	*1.4 ± 0.35*	
	[min, max]	[0.64,1.56]	[0.9,1.21]	[0.59,1.35]	[1.1,1.4]	*[1.1,1.32]*	[0.88,1.37]	[0.98,1.29]	[0.58,1.12]	[1.11,1.33]	*[1.13,2.01]*	n/a
	*p* value	0.92	0.71	0.96	0.26	*0.048*	0.22	0.45	0.98	0.15	*0.015*	
bvFTD *n* = 10	Mean ± sd	1.05 ± 0.15	1.03 ± 0.14	1.01 ± 0.14	1.14 ± 0.17	1.05 ± 0.15	1.05 ± 0.14	1.08 ± 0.16	0.97 ± 0.13	1.12 ± 0.12	1.13 ± 0.13	
	[min, max]	[0.8,1.21]	[0.81,1.23]	[0.8,1.23]	[0.88,1.38]	[0.78,1.25]	[0.81,1.3]	[0.84,1.41]	[0.77,1.2]	[0.91,1.29]	[0.85,1.3]	n/a
	*p* value	0.98	0.48	0.71	0.71	0.25	0.64	0.32	0.35	0.84	0.62	
C9ORF72 & PGRN *n* = 6	Mean ± sd	1.08 ± 0.16	1.08 ± 0.13	1.02 ± 0.16	1.25 ± 0.1	1.12 ± 0.06	1.14 ± 0.1	*1.22 ± 0.15*	1.01 ± 0.11	1.18 ± 0.09	1.2 ± 0.16	
	[min, max]	[0.96,1.39]	[0.95,1.3]	[0.92,1.31]	[1.12,1.41]	[1.06,1.21]	[1.06,1.34]	*[1.01,1.44]*	[0.91,1.22]	[1.07,1.32]	[1.05,1.46]	n/a
	*p* value	0.65	0.98	0.28	0.06	0.96	0.08	*0.039*	0.65	0.24	0.27	
svPPA *n* = 2	Mean ± sd	1 ± 0.11	1.05 ± 0.04	1 ± 0.01	1.2 ± 0.07	1.18 ± 0.07	1.09 ± 0.07	1.11 ± 0.04	0.98 ± 0.08	1.23 ± 0.03	*1.43 ± 0.07*	
	[min, max]	[0.92,1.08]	[1.03,1.08]	[0.99,1.01]	[1.15,1.25]	[1.13,1.23]	[1.04,1.14]	[1.08,1.15]	[0.92,1.04]	[1.21,1.25]	*[1.38,1.47]*	n/a
	*p* value	0.39	0.92	0.53	0.56	0.39	0.65	0.72	0.62	0.09	*0.017*	
Right or IL												
Controls *n* = 53	Mean ± sd	1.05 ± 0.1	1.05 ± 0.1	1.04 ± 0.1	1.17 ± 0.1	1.12 ± 0.12	1.07 ± 0.09	1.1 ± 0.09	1.01 ± 0.09	1.14 ± 0.09	1.13 ± 0.1	1.2 ± 0.16
	[min, max]	[0.78,1.28]	[0.83,1.23]	[0.79,1.28]	[0.78,1.35]	[0.8,1.42]	[0.81,1.28]	[0.85,1.24]	[0.78,1.17]	[0.88,1.27]	[0.88,1.41]	[0.8,1.58]
nfvPPA *n* = 11	Mean ± sd	1.1 ± 0.06	1.1 ± 0.04	*1.08 ± 0.04*	1.2 ± 0.06	1.12 ± 0.05	*1.15 ± 0.04*	*1.18 ± 0.07*	*1.08 ± 0.05*	1.15 ± 0.05	1.11 ± 0.07	
	[min, max]	[1.01,1.17]	[1.01,1.16]	*[1.03,1.16]*	[1.12,1.34]	[1.05,1.24]	*[1.1,1.23]*	*[1.09,1.34]*	*[1,1.17]*	[1.1,1.29]	[1,1.25]	n/a
	*p* value	0.06	0.13	*0.0453*	0.37	0.85	*0.0018*	*0.0029*	*0.0112*	0.96	0.22	
CBS *n* = 7	Mean ± sd	1.04 ± 0.08	1.01 ± 0.06	0.98 ± 0.05	1.18 ± 0.06	1.13 ± 0.04	1.08 ± 0.05	1.1 ± 0.09	1.04 ± 0.05	1.16 ± 0.06	1.08 ± 0.04	1.24 ± 0.1
	[min, max]	[0.94,1.18]	[0.92,1.07]	[0.91,1.04]	[1.06,1.25]	[1.01,1.14]	[1.01,1.14]	[0.97,1.19]	[0.95,1.07]	[1.06,1.22]	[1.02,1.13]	[1.12,1.41]
	*p* value	0.67	0.16	0.049	0.81	0.95	0.88	0.76	0.26	0.48	0.08	0.54
MAPT *n* = 6	Mean ± sd	1.09 ± 0.35	1.08 ± 0.15	1.02 ± 0.25	1.21 ± 0.1	1.21 ± 0.12	1.14 ± 0.19	1.13 ± 0.15	0.95 ± 0.17	1.21 ± 0.08	*1.36 ± 0.35*	
	[min, max]	[0.56,1.49]	[0.91,1.33]	[0.61,1.34]	[1.1,1.32]	[1.09,1.36]	[0.88,1.42]	[0.9,1.35]	[0.62,1.1]	[1.12,1.32]	*[1.1,2.05]*	n/a
	*p* value	0.76	0.82	0.92	0.48	0.15	0.39	0.53	0.73	0.1	*0.035*	
bvFTD *n* = 10	Mean ± sd	1.02 ± 0.12	1.03 ± 0.14	0.98 ± 0.13	1.12 ± 0.13	1.05 ± 0.14	1.05 ± 0.15	1.09 ± 0.14	0.96 ± 0.12	1.11 ± 0.1	1.05 ± 0.07	
	[min, max]	[0.79,1.18]	[0.81,1.2]	0.77,1.14	[0.9,1.31]	[0.79,1.24]	[0.77,1.31]	[0.84,1.31]	[0.76,1.14]	[0.92,1.22]	[0.96,1.15]	n/a
	*p* value	0.61	0.69	0.19	0.28	0.17	0.76	0.51	0.32	0.55	0.007	
C9ORF72 & PGRN n = 6	Mean ± sd	1.06 ± 0.2	1.08 ± 0.14	1.02 ± 0.16	*1.26 ± 0.08*	1.15 ± 0.09	*1.16 ± 0.1*	*1.23 ± 0.15*	1.02 ± 0.11	1.19 ± 0.08	1.17 ± 0.09	
	[min, max]	[0.92,1.44]	[0.98,1.31]	[0.91,1.32]	*[1.17,1.38]*	[1.03,1.24]	*[1.08,1.35]*	*[1.08,1.45]*	[0.94,1.24]	[1.08,1.27]	[1.08,1.29]	n/a
	*p* value	0.46	0.96	0.21	*0.042*	0.54	*0.045*	*0.037*	0.88	0.21	0.62	
svPPA *n* = 2	Mean ± sd	1.03 ± 0.08	1.07 ± 0.01	1 ± 0.1	1.17 ± 0.1	1.13 ± 0.09	1.12 ± 0.08	1.12 ± 0.07	1 ± 0.1	1.16 ± 0.1	1.21 ± 0.05	
	[min, max]	[0.97,1.09]	[1.07,1.08]	[0.99,1.01]	[1.1,1.24]	[1.07,1.19]	[1.06,1.17]	[1.07,1.18]	[0.93,1.07]	[1.09,1.22]	[1.18,1.24]	n/a
	*p* value	0.65	0.75	0.42	0.93	0.96	0.39	0.82	0.89	1	0.15	

*CL* contralateral so symptom onset for CBS group, *CMF* caudal middle frontal, *RMF* rostral middle frontal, *SF* superior frontal, *OFL* orbitofrontal, *IF* inferior frontal, *IN* insula, *PO* pars opercularis, *PT* pars triagularis, *PC* precentral, *TL* temporal lobe, *TP* temporal pole, *WMCMF* white matter caudal middle frontal, *IL* ipsilateral to symptom onset for CBS group

ROIs with significantly elevated signal compared to controls (*p* < 0.05) are italicized

### Corticobasal syndrome

**Fig. 2:**
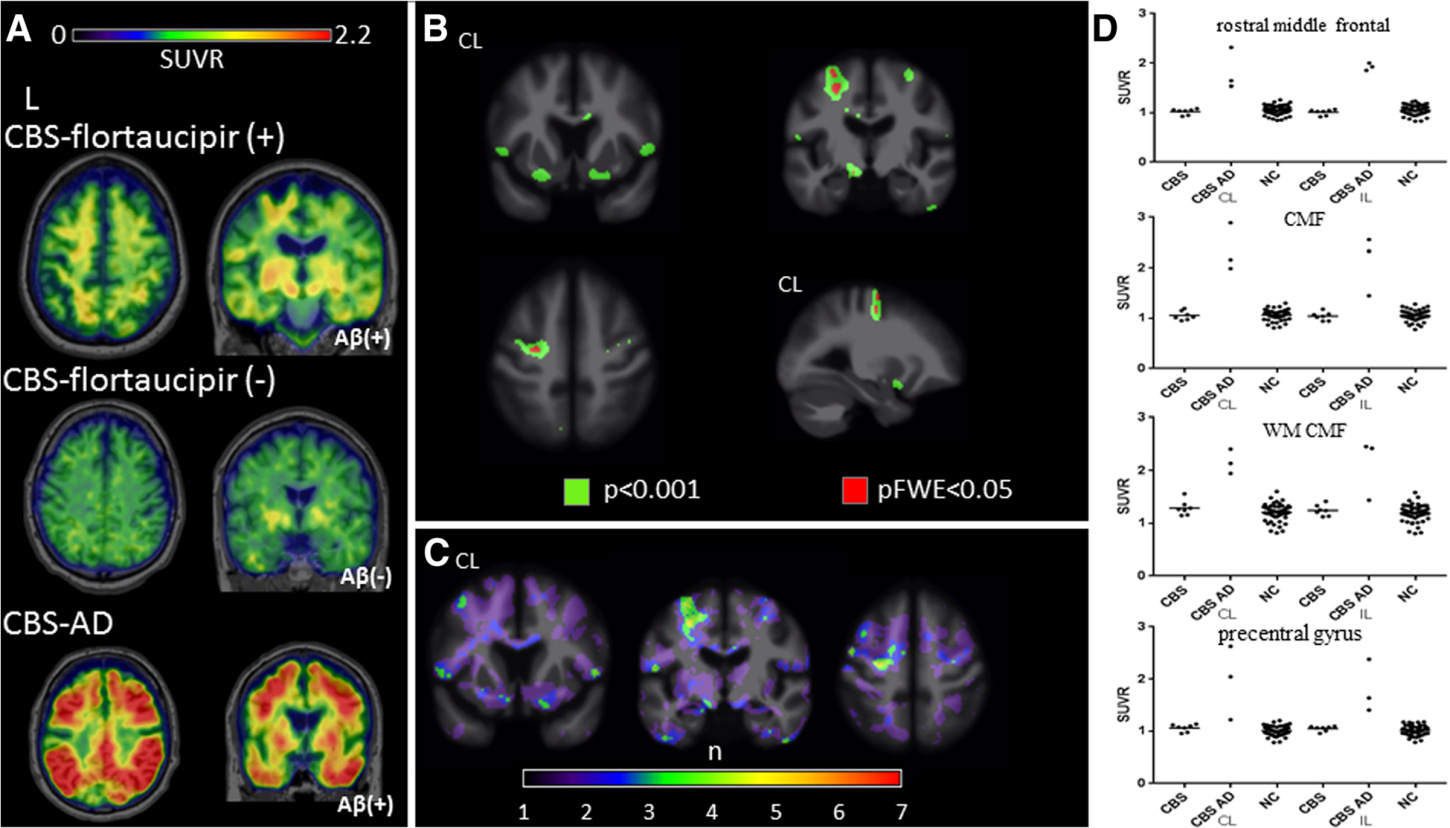
^18^F-flortaucipir in CBS. **a** CBS-flortaucipir (+) example is a 71-year-old female patient, MMSE 29, CDR-SB 2.5, PiB positive. CBS-flortaucipir (−) example is a 60-year-old male patient, MMSE 26, CDR 1.5 PiB negative. CBS-AD example is a 70-year-old male patient, MMSE 25, CDR-SB 7, PiB positive. **b** Voxel-wise contrast of ^18^F-flortaucipir images in seven CBS patients (without CBS-AD images) with 53 normal controls with age as covariate. **c** *W*-score frequency map showing number of patients with suprathreshold age-adjusted SUVR values (*w*-score ≥ 1.65) compared to normal controls. Peak voxels suggest four of seven subjects have elevated *w*-score voxels in the same region. **d** ^18^F-flortaucipir SUVR values for each pre-specified region of interest; horizontal bar denotes mean. NC, normal control; CL, contralateral to symptom onset; IL, ipsilateral to symptom onset; CMF, caudal middle frontal; WM, white matter

^18^F-flortaucipir binding in CBS followed three general patterns shown in Fig. 2a. The first pattern (found in 6/10 patients) included increased uptake in the bilateral frontal cortical gray matter and subcortical white matter, often in the precentral gyrus, superior and middle frontal gyri, hereby referred as CBS-flortaucipir (+). The second, observed in 1/10 patients with CBS, was essentially a null result, with no ^18^F-flortaucipir uptake over background noise, here referred as CBS-flortaucipir (−). The third pattern (3/10), referred to as CBS-AD, was seen only in patients with positive β-amyloid PET, and characterized by high ^18^F-flortaucipir uptake in both degree and extent, similar to levels reported in Alzheimer’s disease. Selected patterns are shown in Fig. 2, and all single-subject scans are shown in Additional file 2: Figure S2. The binding involved bilateral frontal, parietal, temporal, and occipital areas, including the peri-rolandic region, an area spared in typical Alzheimer’s disease. While all CBS-AD patients had positive β-amyloid, the presence of β-amyloid did not necessarily indicate a CBS-AD scan, as two CBS-flortaucipir (+) patients also had positive β-amyloid PET. AI derived from precentral gyrus SUVR to assess whether hemispheric retention differences corresponded to symptom onset laterality demonstrated four of six scans in the CBS-flortaucipir (+) group had higher SUVR in the hemisphere CL to symptom onset meeting the asymmetry ratio cut off. Two of three CBS-AD scans also showed greater uptake in the clinically more affected hemisphere (Additional file 2: Figure S2).

Due to the much higher ^18^F-flortaucupir retention seen in CBS-AD that is suggestive of underlying PHF-tau found in AD rather than the straight or twisted tau filaments seen in CBD, we excluded the three patients in our voxelwise, regional SUVR comparisons to normal controls and *w*-score frequency map generation. Voxelwise comparison demonstrated increased uptake in patients with CBS in the precentral gyrus, middle, inferior frontal gyri, underlying white matter of hemisphere CL to symptom onset and bilateral basal forebrain (*p* < 0.001, uncorrected). Voxels in the middle frontal gyri and white matter CL to symptom onset survived multiple comparisons correction (pFWE< 0.05) (Fig. 2b). *W*-score frequency map demonstrated that approximately half the scans contain voxels with elevated *w*-score above 1.65 in the CL precentral gyrus, middle and inferior frontal gyri (Fig. 2c). A priori regions selected for ROI analyses did not reveal any differences between patients with CBS and controls, though a trend towards increased SUVR uptake was seen in the CL precentral gyrus (*p* = 0.09) (Fig. 2d).

### *MAPT* carriers

**Fig. 3:**
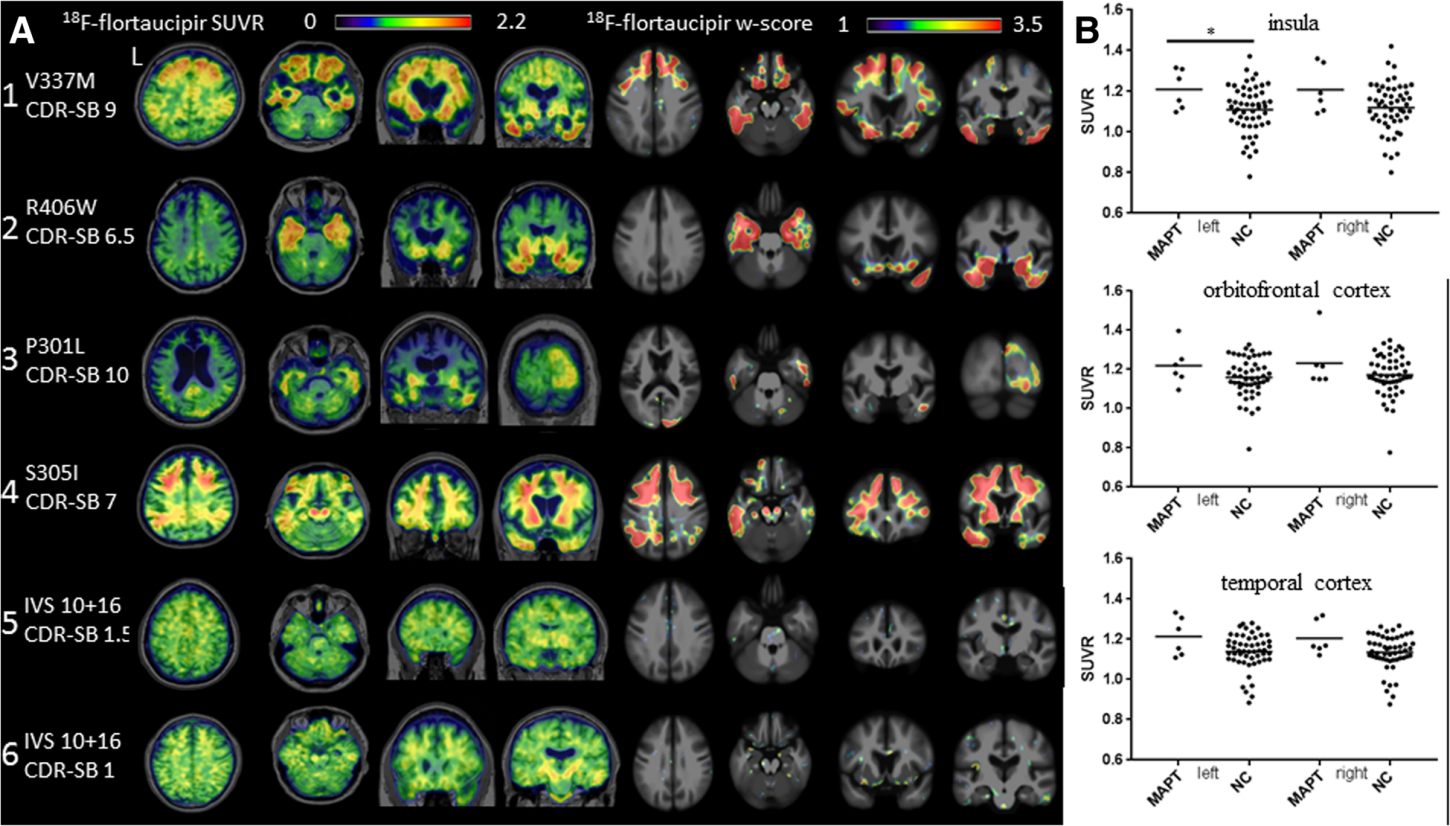
^18^F-flortaucipir in *MAPT* mutation carriers. **a** ^18^F-flortaucipir images and corresponding single-subject *w*-score maps in the following: (1) A 66-year-old V337 M carrier, MMSE 20, CSF Aβ negative. (2) A 68-year-old with R406W mutation, MMSE 16, PiB negative. (3) A 67-year-old P301L carrier, MMSE 5, PiB positive. (4) A 37-year-old S305I carrier, MMSE 4, PiB negative. (5) A 44-year-old IVS10 + 16 carrier, MMSE 30, PiB negative. (6) A 58-year-old IVS10 + 16 carrier, MMSE 27, PiB negative. **b** ^18^F-flortaucipir SUVR values for each pre-specified region of interest; horizontal bar denotes mean. L, left; NC, normal control; CDR-SB, Clinical Dementia Rating scale sum of boxes. *p ≤ 0.05

Six subjects with five distinct *MAPT* mutations with corresponding single-subject *w*-score maps are presented in Fig. 3a. All subjects were ^11^C-PiB negative except patient 3. To protect patient confidentiality, the patients’ sex is omitted. Patient 1, a 66-year-old with V337 M mutation known to cause Alzheimer’s like 3R/4R PHF tau, presented with bvFTD phenotype (CDR-SB 9, MMSE 20). ^18^F-flortaucipir scan showed bilateral frontal, orbitofrontal cortex and anterior, lateral temporal lobe uptake. Patient 2, a 68-year-old with R406W mutation, also associated with 3R/4R PHF tau, presented with amnestic dementia (CDR-SB 6.5, MMSE 16). ^18^F-flortaucipir scan demonstrated uptake in bilateral ventral frontal lobes and widespread retention in the temporal lobes. Patient 3, a 67-year-old with P301L mutation (typically associated with 4R tau) presented with bvFTD (CDR-SB 10, MMSE 5), severe brain atrophy, and a positive ^11^C-PiB scan. ^18^F-flortaucipir scan showed mild uptake in the bilateral temporal and right occipital lobe. Patient 4, a 37-year-old with S305I mutation associated pathologically with 4R tau aggregates resembling AGD [64], presented with behavioral changes and nonfluent aphasia (CDR-SB 7, MMSE 4). ^18^F-flortaucipir scans showed high uptake in bilateral frontal, temporal, parietal lobes and the corresponding white matter. Patients 5 and 6, 44 years old (CDR-SB 1.5, MMSE 30) and 58 years old (CDR-SB 1, MMSE 27) respectively, both carried a splice site mutation (IVS 10 + 16) associated with 4R tau, presented with mild cognitive impairment (MCI). ^18^F-flortaucipir scan for patient 5 demonstrated mild binding in the bilateral basal ganglia, an “off-target” binding region in normal controls, while patient 6 demonstrated low uptake in frontal poles and right lateral temporal lobe. Neither had notable uptake in the single-subject *w*-score maps. A priori ROI SUVR comparison demonstrated tracer binding elevation in the left insula (*p* = 0.048) but not in orbitofrontal or temporal cortex (Fig. 3B), and exploratory regional SUVR comparison showed increased uptake in bilateral temporal poles (left *p* = 0.015, right *p* = 0.035) (Table 2).

### Sporadic behavioral variant frontotemporal dementia

**Fig. 4:**
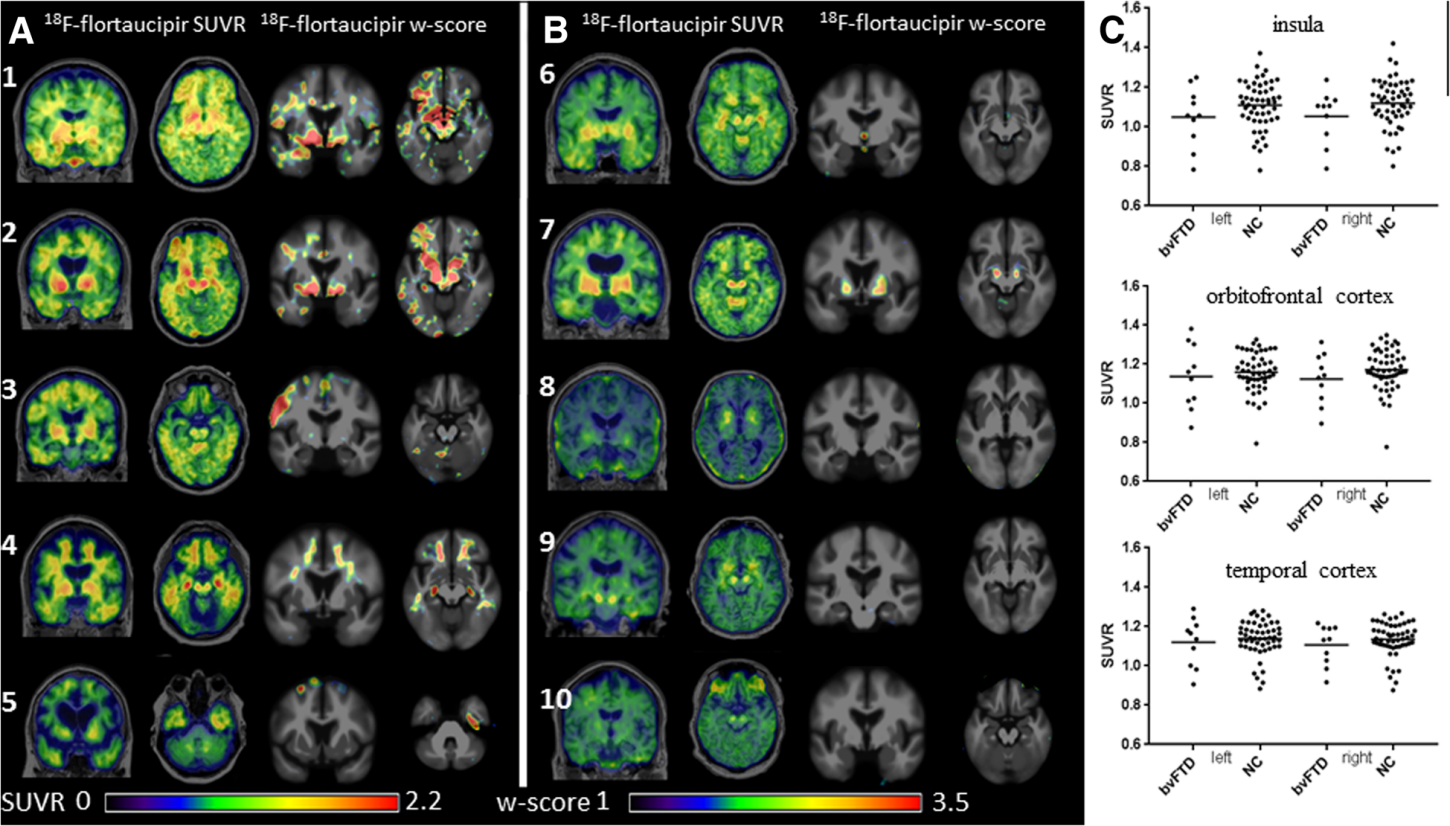
^18^F-flortaucipir in bvFTD. **a** Five bvFTD scans and corresponding single-subject *w*-score maps determined to have clear uptake signal in frontal and temporal lobes. Patient 1 is a 46-year-old male, MMSE 22, CDR-SB 4, PiB negative. Patient 2 is a 54-year-old female, MMSE 19, CDR-SB 5, PiB negative. Patient 3 is a 74-year-old male with MMSE 23, CDR-SB 7.5, PiB positive. Patient 4 is a 69-year-old male, MMSE 22, CDR-SB 9, PiB positive. Patient 5 is a 78-year-old female, MMSE 18, CDR-SB 12, PiB positive. **b** Five bvFTD scans and corresponding single-subject *w*-score maps determined to have minimal or no uptake in frontal and temporal lobes. Patient 6 is a 76-year-old male, MMSE 26, CDR-SB 6.5, PiB negative. Patient 7 is a 74-year-old male, MMSE 26, CDR-SB 1.5, PiB negative. Patient 8 is a 64-year-old male, MMSE 27, CDR-SB 7.5, PiB negative. Patient 9 is a 68-year-old male, MMSE 29, CDR-SB 5, PiB negative. Patient 10 is a 34-year-old male, MMSE 22, CDR-SB 8, Aβ status unavailable. **c** ^18^F-flortaucipir SUVR values for each pre-specified region of interest; horizontal bar denotes mean. L, left; NC, normal control

All patients with sporadic bvFTD presented with behavioral changes without motor neuron disease. Patients 3–5 had positive ^11^C-PiB PET imaging thus only fulfilling possible bvFTD criteria, while patient 10 did not undergo β-amyloid testing but was unlikely to have β-amyloid based on young age of 34. ^18^F-flortaucipir images were classified as positive or negative for significant frontal or temporal lobe uptake based on qualitative and quantitative assessments utilizing *w*-score maps. Five (three with positive β-amyloid status) were determined to have elevated frontal or temporal uptake (Fig. 4a) while five were determined to have no clear binding in frontotemporal regions (Fig. 4b). No patients showed binding typical of the Alzheimer’s disease range (Additional file 3 Figure S3). ROI SUVR comparison did not demonstrate differences from controls at the group level (Fig. 4c).

### *C9ORF72*, *GRN* mutation carriers, and semantic variant primary progressive aphasia

**Fig. 5:**
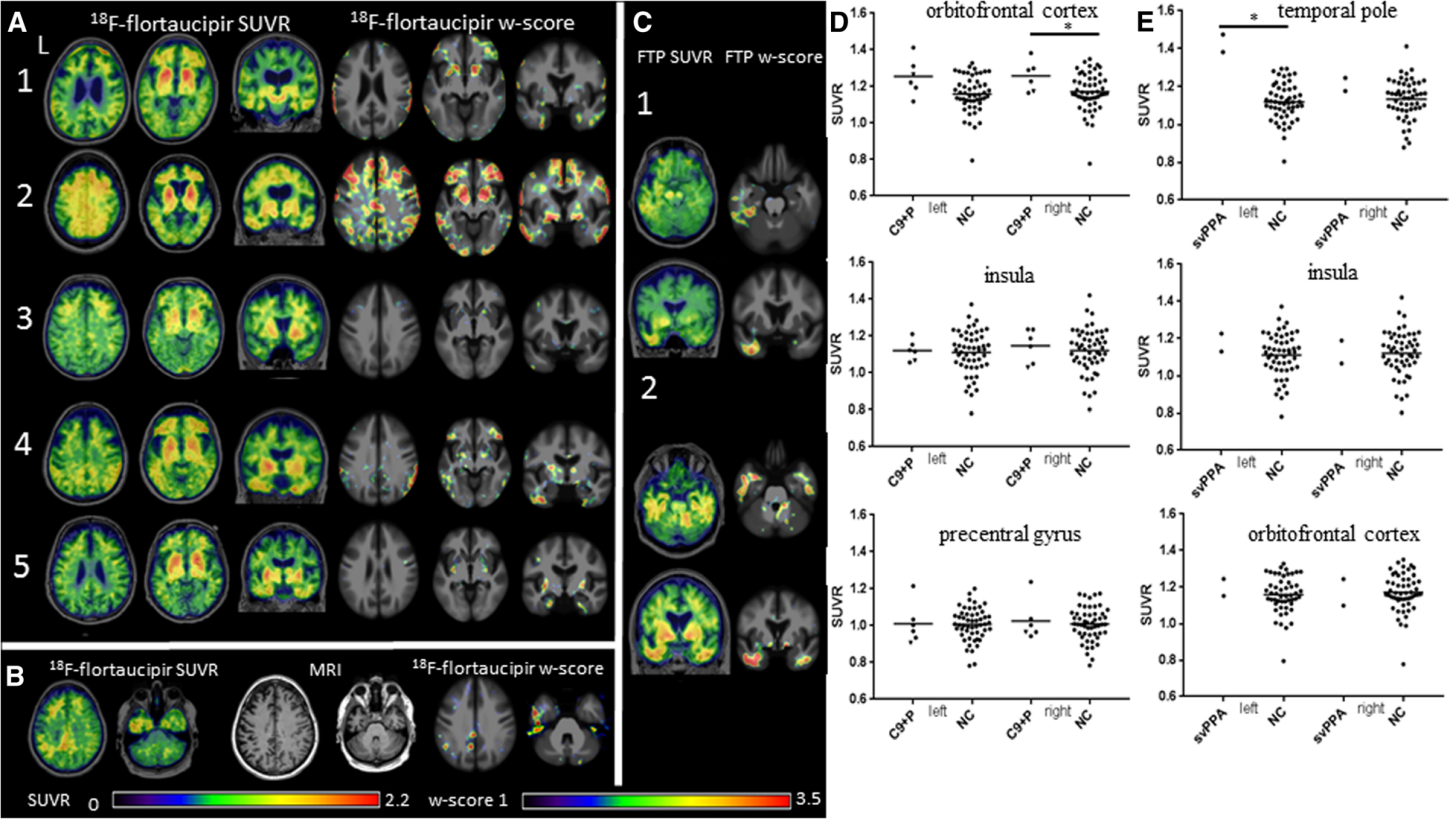
^18^F-flortaucipir in *C9ORF72*, *GRN* mutation carriers and svPPA. **a** Five *C9ORF72* carriers scanned with ^18^F-flortaucipir and corresponding single-subject *w*-score maps. Patient 1 is 71 years old, MMSE 26, CDR-SB 1, PiB negative. Patient 2 is 67 years old, MMSE 22, CDR-SB 8, Aβ status unavailable. Patient 3 is 48 years old, MMSE 26, CDR-SB 7, PiB negative. Patient 4 is 54 years old, MMSE 26, CDR-SB 7, PiB negative. Patient 5 is 71 years old, MMSE 26, CDR-SB 0.5 PiB negative. **b** *GRN* mutation carrier with corresponding structural MRI and single-subject *w*-score map, 55 years old, MMSE 21, CDR-SB 4.5, PiB positive. Structural MRI demonstrates asymmetrical left hemispheric atrophy corresponding to location of tracer uptake. **c** Two svPPA patients with corresponding single-subject *w*-score maps. Patient 1 is a 59-year-old female, MMSE 28, CDR-SB 3.5, PiB negative. Patient 2 is a 71-year-old male, MMSE 25, CDR-SB 3.5, PiB negative. **d** ^18^F-flortaucipir SUVR values for patients with *C9ORF72* and *GRN* mutations (C9 + P), for each pre-specified region of interest. **e** ^18^F-flortaucipir SUVR values for patients with svPPA for each pre-specified region of interest; horizontal bar denotes mean. L, left; NC, normal control; FTP, ^18^F-flortaucipir; *p ≤ 0.05

All *C9ORF72* carriers presented with clinical bvFTD except patient 5 who presented with motor neuron disease and executive dysfunction. No β-amyloid biomarker results were available for patient 2, who developed new memory and visual spatial symptoms 2 years after ^18^F-flortaucipir imaging, concerning for Alzheimer’s disease. All remaining patients were β-amyloid negative. Overall, varying degrees of ^18^F-flortaucipir uptake were seen in the frontal poles in all patients though not all uptake are present in single-subject *w*-score maps. Patient 2 displayed additional binding throughout the frontoparietal gray, white matter and bilateral temporal lobes. Patient 3 demonstrated mild uptake in the frontal poles, anterior middle frontal gyrus, left greater than right inferior temporal regions that were negligible in the *w*-score map, while patient 4 demonstrated uptake in the bilateral frontal poles, medial and inferior temporal lobes as well as parietal cortex. Patient 5 had the least tracer retention in the frontal poles, with additional binding in the bilateral medial and inferior temporal lobes (Fig. 5a). A patient with a *GRN* mutation and positive ^11^C-PiB presented with memory, visual spatial, and behavioral dysfunction. ^18^F-flortaucipir scan demonstrated elevated uptake in the left lateral frontal, parietal and temporal lobes, corresponding to asymmetric atrophy seen on MRI though frontal lobe uptake was absent on corresponding *w*-score map (Fig. 5b). ^18^F-flortaucipir images of two patients with svPPA demonstrated uptake restricted to the left anterior temporal pole in the first, and bilateral orbital frontal lobe and temporal lobe in the second (Fig. 5c). A priori ROI SUVR comparison in the *C9ORF72* and *GRN* carriers showed elevated uptake in the right orbitofrontal cortex (*p* = 0.042) (Fig. 5d), but additional exploratory regions demonstrated elevations in right pars opercularis (*p* = 0.045), and bilateral pars triangularis (left *p* = 0.039, right *p* = 0.037) (Table 2). ROI comparison in svPPA demonstrated elevated uptake in the left temporal pole only (*p* = 0.017) (Fig. 5e).

### Autopsy results

Patient 3 in the *C9ORF72* cohort was 48 years old with 5 years of executive dysfunction, behavioral disinhibition, eating compulsions, and lack of empathy. Family history was notable for ALS in multiple family members. Neurological exam noted overt jocularity, emotional disconnection, while neuropsychological testing demonstrated executive and verbal memory dysfunction. ^11^C-PiB PET was negative. The patient died 21 months after PET. At autopsy the patient met neuropathological criteria for FTLD-TDP, Type B. Microvacuolation and gliosis could not be evaluated due to severe dehydration artifact caused during postmortem handling. Tau immunostaining with CP-13 detected mild, likely incidental tau co-pathology of two types. First, there was Braak stage 1 neurofibrillary pathology, with additional scattered tangle and thread pathology in the middle frontal gyrus, inferior temporal gyrus gray matter, and amygdala. Second, there was aging-related tau astrogliopathy (ARTAG), possibly consistent with the effects of remote head trauma, in the inferior temporal gyrus and amygdala. ^18^F-flortaucipir image demonstrated mild uptake in the bilateral anterior middle frontal gyrus, underlying white matter and left greater than right inferior temporal gyrus. TDP-43 immunohistochemistry detected large numbers of inclusions in the frontal pole, middle frontal gyrus, cingulate cortex, inferior temporal gyrus, amygdala, entorhinal cortex, and additional deposits were found in the precentral gyrus, anterior horn cells of the spinal cord and substantia nigra. β-amyloid immunostaining observed sparse plaques in the angular gyrus and striate cortex. Ubiquitin immunohistochemistry identified p62-positive, TDP-43 negative stellate/round neuronal cytoplasmic inclusions in cerebellar granule cells, consistent with *C9ORF72* mutation.

**Fig. 6:**
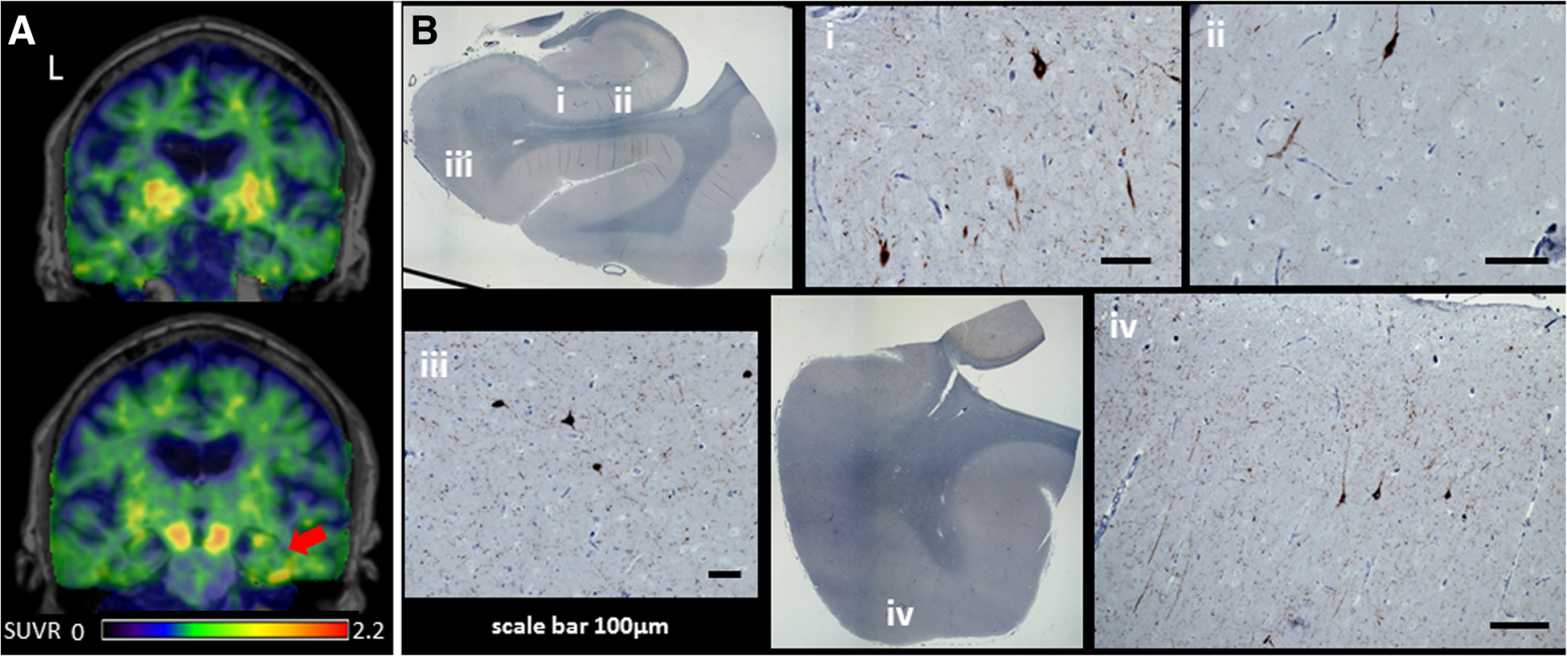
Positron emission tomography (PET) to autopsy comparisons. **a** ^18^F-flortaucipir standardized uptake value ratio (SUVR) images shown alongside corresponding histological slides for a 68-year-old man with behavioral variant frontotemporal dementia due to argyrophilic grain disease (AGD), a 4-repeat tauopathy and Braak Alzheimer’s disease stage 3. The red arrow denotes areas of signal uptake. **b** Histological slides across the hippocampus at the level of the lateral geniculate body immunostained for phospho-tau protein (Ser202, CP-13, 1:500, gift of Peter Davies). (i) CA1 sector showing abundant grains and few neurofibrillary tangles. (ii) CA 1 sector showing abundant grains and few neurofibrillary tangles. (iii) Subiculum showing grains and pretangles. (iv) Inferior temporal gyrus showing grains and pretangles. L, left

Patient 9 of the bvFTD cohort was a 68 year old right handed male with past medical history significant for fascioscapulohumeral dystrophy with motor symptoms since age 20. He presented with 7 years of hyper-orality, executive dysfunction, hyper sexuality and obsessive behaviors. Neurological exam identified bilateral facial, upper, lower extremity weakness greater in the proximal muscles, and neuropsychological testing demonstrated deficits in memory, visual spatial, and executive functions. ^11^C-PiB was negative. The patient died 3 months after PET due to progressive muscular dystrophy. At autopsy, the patient met criteria for AGD. Tau immunohistochemistry analysis showed right greater than left severe neuropil thread/grain pathology in the anterior entorhinal cortex, CA1/subiculum, and parahippocampal gyrus, with milder deposits seen in anterior orbital gyrus and cingulate cortex. Moderate to severe NFT pathology was also found in the amygdala, CA1/subiculum, and entorhinal cortex. ^18^F-flortaucipir scan demonstrated a small area of retention in the right greater than left inferior temporal gyrus and portions of the hippocampus (Fig. 6a). Tracer binding was not found in the entorhinal cortex. No other areas of cortical uptake were seen. Neuropathology examination further revealed right greater than left hemisphere gliosis and microvacuolation mostly in the inferior temporal gyrus, entorhinal cortex, amygdala, and CA1/subiculum (Fig. 6b). β-amyloid, ubiquitin, alpha synuclein, and TDP-43 immunohistochemistry were unremarkable.

## Discussion

In the present study, we describe ^18^F-flortaucipir PET findings in an extended series of patients covering the FTD clinical and genetic spectrum. Overall, on a review of both SUVR and single-subject *w*-score maps, we observed patterns of low-level ^18^F-fluortaucipir binding that closely matched the expected anatomical distribution and frequency of tau pathology in the tau-associated FTD syndromes nfvPPA, CBS, and a subset of bvFTD. Tracer retention was low though single-subject *w*-score maps often supported visually assessed ^18^F-flortaucipir images with the presence of voxels with significant retention compared to controls. However, ROI comparisons with controls frequently did not demonstrate regions with significant retention or showed considerable overlap between patients and controls. Distinct binding patterns were seen in CBS, possibly differentiating CBS due to underlying Alzheimer’s disease versus FTLD pathology. In *MAPT* mutation carriers, tracer uptake was seen primarily (though not exclusively) in mutations with Alzheimer’s disease-like tangles. In two patients with PET to autopsy correlation, mild ^18^F-flortaucipir binding was seen in some areas with tau pathology (NFT or AGD), but binding patterns did not correspond with the distribution of FTLD TDP-43 type B inclusions. Overall, the degree of tracer binding in non-Alzheimer’s tauopathies was considerably lower than seen in Alzheimer’s disease, though was qualitatively distinct from binding in β-amyloid negative normal controls. These results are consistent with low-affinity ^18^F-fluortaucipir binding to at least a subset of tau aggregates in these disorders, or alternatively to a process that co-localizes with tau pathology. Furthermore, notable tracer uptake in syndromes and mutations associated with TDP-43 pathology raises concerns about the specificity of ^18^F-flortaucipir binding for FTLD tau pathology.

### ^18^F-flortaucipir uptake matched expected distribution, frequency of tau pathology in nfvPPA, bvFTD, and CBS

Clinicopathological series of patients with nfvPPA show the predominant underlying pathology to be FTLD-tau [3]. In our case series, all nfvPPA images displayed ^18^F-flortaucipir uptake in the inferior frontal regions, covering the frontal operculum (Additional file 1: Figure S1). Our series also reflected the heterogeneous neuropathology underlying other FTD syndromes. In bvFTD, an autopsy series of 117 patients demonstrated 34 and 55 cases of FTLD-tau, FTLD-TDP respectively [4]. In our series, a bimodal separation was seen qualitatively where five of ten patients with bvFTD showed frontotemporal tracer uptake, possibly reflecting the differentiation between tau and TDP. No ROI SUVR differences or quantitative voxelwise comparisons were demonstrated, likely due to the heterogenous degree of binding seen within the group.

The underlying pathology in patients presenting clinically with CBS is heterogeneous, half with underlying 4R tauopathy (CBD or PSP), approximately 25% showing primary Alzheimer’s disease, and a minority with FTLD-TDP [2, 65]. Qualitative assessment on SUVR review showed six of ten patients with CBS here demonstrated tracer uptake in the precentral gyrus and frontal white matter, areas rich in CBD tau pathology, though *w*-score map review suggest tracer uptake in patient 6 does not reach > 95 percentile of normal distribution threshold compared to normal controls (Additional file 2: Figure S2). Four of these six patients showed asymmetric binding in precentral gyrus of hemisphere CL to symptom onset, a finding also present in two of three CBS-AD patients. Both qualitative and quantitate analysis suggest the ^18^F-flortaucipir(−) patient had minimal to no cortical or white matter uptake, which may suggest this is CBS with FTLD-TDP. However, ^18^F-flortaucipir retention seen in svPPA, TDP-43 associated mutation carriers makes this inference tenuous. Our findings were similar to a recent report in six β-amyloid negative patients with CBS with elevated precentral white matter ^18^F-flortaucipir binding that correlated with motor severity [20], while another series reported six of eight patients with CBS with asymmetric ^18^F-flortaucipir binding in motor cortex and white matter [21].

### ^18^F-flortaucipir shows preferential binding to specific tau species

^18^F-flortaucipir was developed by screening tracer binding to postmortem tissue rich in Alzheimer’s disease NFT, composed of 3R and 4R tau forming PHF [13]. Conversely, tau aggregates in FTLD primarily consist of 4R (CBD, PSP) or 3R tau aggregating as straight or twisted filaments. Autoradiography studies have reported absent to low-affinity ^18^F-flortaucipir binding to non-Alzheimer’s disease aggregates, depending on tissue preparation protocols and other procedures [14, 15, 32]. Similar to our previous report in PSP and cases series from other groups, we generally find low-level binding in non-Alzheimer’s disease tauopathies. The SUVR values in regions of expected tau pathology are lower than those reported in Alzheimer’s disease [61], but at a group level higher than those observed in β-amyloid negative normal controls, and can be distinguished qualitatively based on regional uptake patterns. In a patient with sporadic bvFTD and underlying AGD pathology (a 4R tauopathy), ^18^F-flortaucipir detected only areas with the highest concentration of tau pathology. Overall, these findings are consistent with low affinity, rather than absent tracer binding to non-Alzheimer’s disease tau.

Binding in *MAPT* mutation can be particularly enlightening given the heterogeneous but well-described characteristics of tau associated with particular mutations. Consistent with previous reports [19, 66], we found the highest ^18^F-flortaucipir uptake in patients carrying V337 M and R406W mutations, associated with “Alzheimer’s-like” tau aggregates of 3R/4R isoforms aggregated as PHF. In contrast, as reported by others [66, 67], lower binding was seen in P301L and IVS10 + 16 mutations associated with 4R tau aggregates composed of straight or twisted tau filaments [8]. An important caveat regarding the IVS10 + 16 mutation is that participants were at an early symptomatic stage. Overall, these distinctions across *MAPT* mutations are consistent with the notion that ^18^F-flortaucipir binds with highest affinity to biochemically and microstructurally “Alzheimer’s-like” tau tangles. However, to every rule there is an exception. We report for the first time (to our knowledge) extensive tracer binding in a patient with S305I mutation, associated with a pure 4R tauopathy resembling AGD and straight filament structure [64]. Therefore, simple heuristics based on tau isoforms or filament type may not fully capture the nuances and complexity of tracer interaction with the heterogeneous spectrum of tau pathology.

### Postmortem analysis showed partial correspondence of ^18^F-flortaucipir binding to FTLD-tau and not FTLD-TDP

Recent imaging to autopsy evidence further supports weak but present binding of ^18^F-flortaucipir to tau inclusions seen in FTLD-tau. Recent case reports of patients with corticobasal degeneration showed regional in vivo ^18^F-flortaucipir SUVR correlation with tau burden at autopsy [22, 68]. Autopsy examination 9 months after ^18^F-flortaucipir imaging of a PSP Richardson’s syndrome patient revealed severe corticobasal degeneration pathology in frontal, perirolandic, posterior cingulate regions and subcortical regions of globus pallidus, striatum, thalamus, subthalamic nucleus, midbrain, pons, and dentate nucleus that corresponded to ^18^F-flortaucipir binding. Of note, tau pathology was observed in insula and postcentral gyrus which did not demonstrate increased ^18^F-flortaucipir binding [28]. Here in a symptomatic *C9ORF72* expansion carrier, tau tracer uptake was mild, not present in *w*-score map cut off, and may reflect the mild, scattered tau co-pathology, though the sparse deposits broach the possibility of additional co-localized targets responsible for the higher than background signal. No retention was seen in areas of solely FTLD-TDP Type B pathology such as the precentral gyrus and cingulate cortex. In the second autopsy patient, focal ^18^F-flortaucipir retention corresponded to an area of severe AGD. However, NFT pathology in close proximity and limited PET resolution suggests AGD may not be the sole source of tracer binding. The relatively weak retention in relation to the uptake seen in normal controls did not allow the authors to determine the PET image to be “positive” when blinded to autopsy results. Thus, we provide more evidence that ^18^F-flortaucipir does not bind to TDP-43, but its weak retention in certain FTLD-tau subtypes and still uncertain specificity may limit diagnostic utility.

### ^18^F-flortaucipir may help differentiate FTD due to Alzheimer’s pathology from incidental β-amyloid co-pathology

While the majority of FTD syndromes are caused by tau or TDP-43, a subset have extensive Alzheimer’s disease pathology, especially in CBS [2]. β-amyloid biomarkers may help identify underlying Alzheimer’s disease in patients with clinical FTD. However, β-amyloid pathology is also found in a sizable minority of cognitively normal individuals and patients with dementia, reducing the positive predictive value of β-amyloid PET in older adults [69]. Tau imaging, in combination with β-amyloid PET, may help discriminate incidental β-amyloid pathology from true underlying Alzheimer’s disease in these complex scenarios, since the pattern and intensity of ^18^F-flortaucipir binding differentiate Alzheimer’s disease from non-Alzheimer’s disorders [70]. In CBS, three of ten ^11^C-PiB-positive patients demonstrated a pattern and degree of ^18^F-flortaucipir uptake highly suggestive of Alzheimer’s disease. This mirrors a recent report where two of eight β-amyloid positive CBS patients demonstrated prominent uptake in the temporoparietal lobes [21]. Of note, positive β-amyloid status is necessary but not sufficient for a CBS-AD type scan. Patients 1 and 6 in our series had positive ^11^C-PiB scans and demonstrated asymmetric uptake in frontal cortex and white matter, with SUVRs well below those seen in Alzheimer’s disease (Additional file 2: Figure 2). Three bvFTD patients (patients 3–5) were ^11^C-PiB positive yet presented with SUVRs well below those seen in Alzheimer’s disease. This argument is further strengthened when we compare all FTD cases against a group of age, sex, disease severity matched patients with Alzheimer’s disease. In the temporal region, only the three CBS-AD cases had SUVR comparable to Alzheimer’s disease. Furthermore, in the precentral gyrus, an area of late pathological involvement in Alzheimer’s, the CBS-AD SUVR were at the highest range compared to those seen in Alzheimer’s disease (Additional file 3: Figure S3). We speculate that ^18^F-flortaucipir may help differentiate FTD spectrum disorders due to Alzheimer’s disease from those with primary FTLD pathology and incidental or preclinical β-amyloid pathology.

### ^18^F-flortaucipir may reflect disease onset in *MAPT*

In our qualitative assessment of *MAPT* carriers, low tracer uptake was seen in two IVS 10 + 16 carriers diagnosed with MCI, while more prominent, widespread uptake was seen in *MAPT* carriers with dementia. Contrary to familial Alzheimer’s disease where β-amyloid PET retention is evident a decade or longer before symptom onset, ^18^F-flortaucipir imaging may turn positive more proximate to symptom onset in *MAPT* carriers. This is supported by the minimal neocortical tau pathology seen in a patient with *MAPT* mutation at + 3 intron 10 and only one year of symptoms [71]. An important caveat to this interpretation is that we did not have asymptomatic and symptomatic patients with the same mutation for comparison.

### ^18^F-flortaucipir binding seen in patients with predicted TDP-43 proteinopathy

Previous autoradiographic evidence suggested absent or minimal binding to TDP-43 pathology [15]. ^18^F-flortaucipir tracer uptake was seen in varying degrees in patients with predicted TDP-43 here. svPPA is nearly always associated with TDP-43 pathology with initial neurodegeneration in the temporal poles [58], and both patients in our series demonstrated left greater than right temporal pole tracer uptake with elevated ROI SUVR, similar to recent reports [29, 30]. The *GRN* mutation carrier also showed asymmetric tracer binding, corresponding to asymmetric cortical atrophy. Complicating interpretation is the fact that this patient had positive ^11^C-PiB, raising the possibility that ^18^F-flortaucipir retention is detecting early Alzheimer’s disease-related tau.

In *C9ORF72* mutations, orbitofrontal, inferior temporal, anterior insular, cingulate, cerebellar, hippocampus and thalamic atrophy is often seen, with TDP-43 aggregates in neuroanatomical regions including the extramotor cerebral cortex, hippocampus and basal ganglia [54, 57, 72, 73]. All *C9ORF72* carriers here presented with varying degrees of tracer binding, especially in frontal poles and inferior temporal lobes. Previous clinicopathological correlations suggest more TDP-43 pathology in the frontotemporal neocortex in patients with FTD than those with motor neuron disease alone [9]. Patient 5, who presented with ALS and only executive dysfunction, also demonstrated the least frontal binding. While tau pathology has been reported in both *C9ORF72* and *GRN* mutation carriers (and was found on autopsy in one of the carriers here), it is difficult to conclude that ^18^F-flortaucipir retention in *C9ORF72* carriers is caused by tau [74, 75]. Based on the overlap between tracer uptake and areas of atrophy, ^18^F-flortaucipir may be binding to non-tau targets of neurodegeneration. For example, binding to neuromelanin- containing cells, monoamine oxidase-A, calcified structures or iron deposits have been demonstrated to varying degrees [14, 31, 76]. However, the lack of tracer uptake in some sporadic bvFTD and CBS patients with marked atrophy on MRI argues against ^18^F-flortaucipir being an entirely non-specific marker of neurodegeneration.

### Potential applications of ^18^F-flortaucipir

A more sensitive and specific tracer for non-Alzheimer’s disease tauopathies is desirable, and given the multiplicity of tau conformations, multiple tracers may be needed. While efforts are underway, given the rapid pace of tauopathies treatment development, there are several potentially useful applications for ^18^F-flortaucipir in FTD currently. When tau pathology can be confidently predicted based on clinical syndrome or genetic mutation, longitudinal ^18^F-flortaucipir images may provide an understanding of how tau spreads *vis-a-vis* clinical progression and other biomarker changes. Longitudinal ^18^F-flortaucipir may also serve as a potential pharmacodynamic biomarker for tau-treatments in development, where decreasing uptake compared to placebo suggests target engagement [11]. This may improve the efficiency of clinical trials. Furthermore, the combination of β-amyloid and ^18^F-flortaucipir PET could help differentiate FTD due to Alzheimer’s disease from incidental β-amyloid pathology in patients with primary FTLD pathology. This will provide a more accurate prediction of underlying neuropathology during life, but may also aid future clinical trials if treatments that target specific tauopathies are developed.

Strengths of this study include the relatively large series of patients who cover a broad spectrum of FTD syndromes and genetic mutations. In addition, we add to the literature two patients with in vivo ^18^F-flortaucipir and postmortem comparisons, highlighting the potential and also limitations of the tracer in detecting FTLD. A limitation of this study is the lack of a consensus standardized visual classification scheme for Alzheimer’s disease and FTD ^18^F-flortaucipir scans due to the tracer’s relatively new availability. The absence of postmortem autopsy in all patients, especially given complex in vivo results, also limits our interpretation. Also, our region-of-interest SUVR comparison often failed to demonstrate differences to normal controls, likely as a result of either the heterogeneous underlying pathology in FTD resulting in a range of tracer retention within syndromes, or that the region-of-interest mask surpassed binding area, resulting in lowering of averaged binding strength.

## Conclusions

In a series of patients with FTD syndromes and mutation carriers imaged with ^18^F-flortaucipir, uptake was seen in expected areas of tau pathology in nfvPPA, CBS, bvFTD and *MAPT* carriers, with the frequency of positive scans among these diagnostic cohorts reflecting the heterogeneous neuropathology of FTD. Elevated binding in neurodegenerative disease with predicted TDP-43 pathology raises questions about the specificity of the tracer. Further studies with postmortem comparisons will be essential to understand the complex and nuanced in vivo findings noted across centers when applying ^18^F-flortaucipir in FTD. Despite potential utility of ^18^F-flortaucipir PET proposed here, more sensitive and specific tracers will be needed to optimally capture FTD tau pathology.

## Additional files

Additional file 1: **Figure S1.** ^18^F-flortaucipir in nfvPPA ^18^F-flortaucipir images and corresponding single-subject *w*-score map in all 11 patients diagnosed with nfvPPA. (TIF 814 kb)

Additional file 2: **Figure S2.** ^18^F-flortaucipir in CBS ^18^F-flortaucipir images and corresponding single-subject *w*-score map in patients diagnosed with CBS and corresponding β-amyloid status determined via PiB imaging. Numerical value indicates laterality of asymmetric index (AI) defined as 200 × (right uptake-left uptake)/(right uptake + left uptake) of SUVR in precentral gyrus, with a minimum threshold of 1.91 and laterality of symptom onset determined by an asterisk. (TIF 739 kb)

Additional file 3: **Figure S3.** ^18^F-flortaucipir in FTD, NC and AD ^18^F-flortaucipir SUVR in temporal and precentral gyrus across all FTD syndromes, normal controls and a cohort (*n* = 45) of age (median, min, max) (63, 48, 77), sex (21 female, 24 male), Mini-Mental State Examination [4, 24, 30], Clinical Dementia Rating scale sum of boxes (4, 0.5, 7) matched Alzheimer’s disease patients. For all 4 regions of interest, AD and CBS-AD group had higher SUVR compared to nfvPPA, CBS, MAPT, bvFTD, C9ORF72 & PGRN, svPPA and normal controls.(*p* < 0.05). No differences between AD and CBS-AD across all 4 regions of interest. CL, contralateral; IL, ipsilateral; horizontal bar denotes mean. (TIF 65 kb)

## Abbreviations

AD: Alzheimer’s disease
AGD: Argyrophilic grain disease
ARTAG: Aging-related tau astrogliopathy
bvFTD: Behavioral variant frontotemporal dementia
C9ORF72: Chromosome 9 open reading frame 72 gene
CBD: Corticobasal degeneration
CBS: Corticobasal syndrome
FTD: Frontotemporal dementia
FTD-ALS: Frontotemporal dementia with amyotrophic lateral sclerosis
FTLD: Frontotemporal lobar degeneration
GGT: Globular glial tauopathy
GRN: Progranulin
MAPT: Microtubule-associated protein tau
MRI: Magnetic resonance imaging
NFT: Neurofibrillary tangle
nfvPPA: Nonfluent variant primary progressive aphasia
PET: Positron emission tomography
PHF: Paired helical filament
PSP: Progressive supranuclear palsy
SUVR: Standardize uptake value ratio
svPPA: Semantic variant primary progressive aphasia
TDP-43: TAR DNA-binding protein
