# Supplementary figures and images for: 18F-flortaucipir (AV-1451) tau PET in frontotemporal dementia syndromes
Source: Alzheimers Res Ther. 2019 Jan 31;11:13. doi: 10.1186/s13195-019-0470-7 (PMC6357510; doi:10.1186/s13195-019-0470-7)

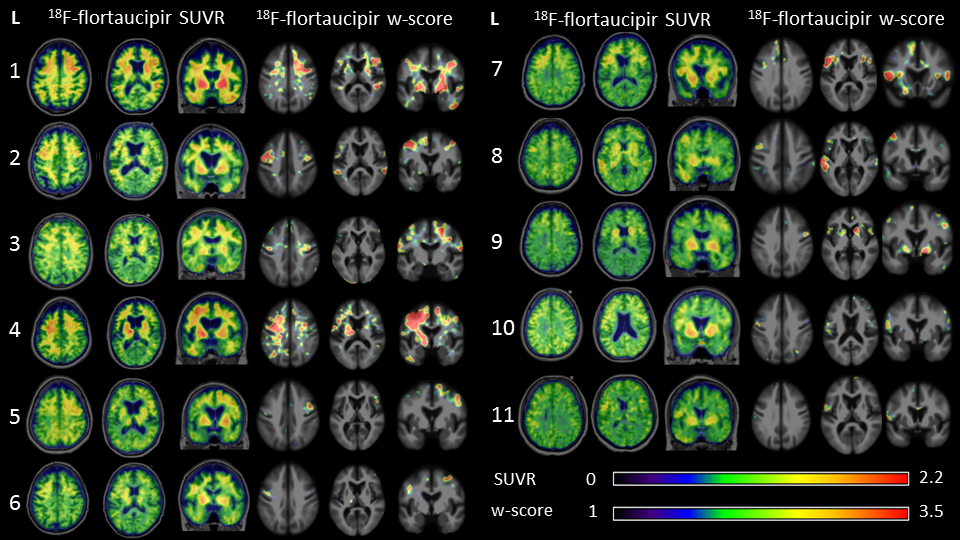

Supplement: Supplementary file 1 — Figure S1. 18F-flortaucipir in nfvPPA 18F-flortaucipir images and corresponding single-subject w-score map in all 11 patients diagnosed with nfvPPA. (TIF 814 kb) [file 13195_2019_470_MOESM1_ESM.tif]

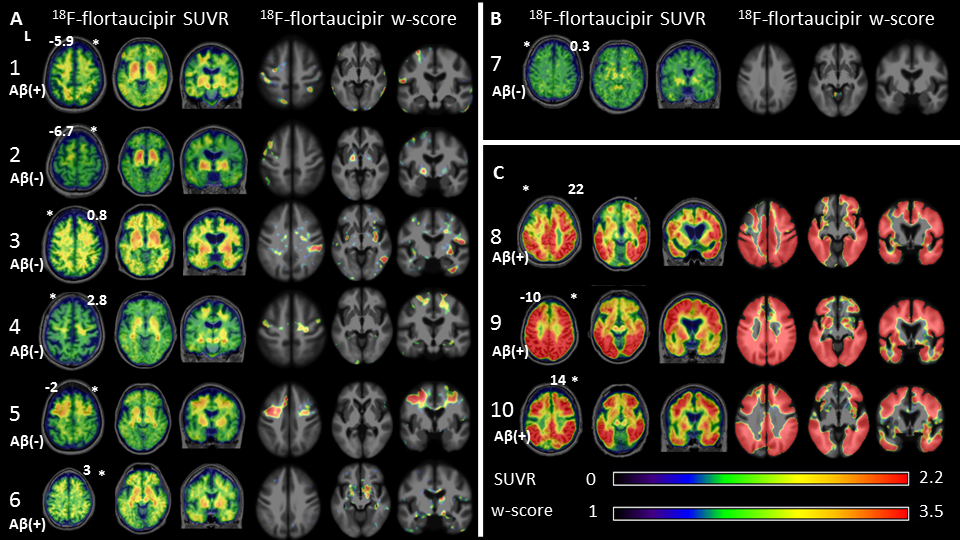

Supplement: Supplementary file 2 — Figure S2. 18F-flortaucipir in CBS 18F-flortaucipir images and corresponding single-subject w-score map in patients diagnosed with CBS and corresponding β-amyloid status determined via PiB imaging. Numerical value indicates laterality of asymmetric index (AI) defined as 200 × (right uptake-left uptake)/(right uptake + left uptake) of SUVR in precentral gyrus, with a minimum threshold of 1.91 and laterality of symptom onset determined by an asterisk. (TIF 739 kb) [file 13195_2019_470_MOESM2_ESM.tif]

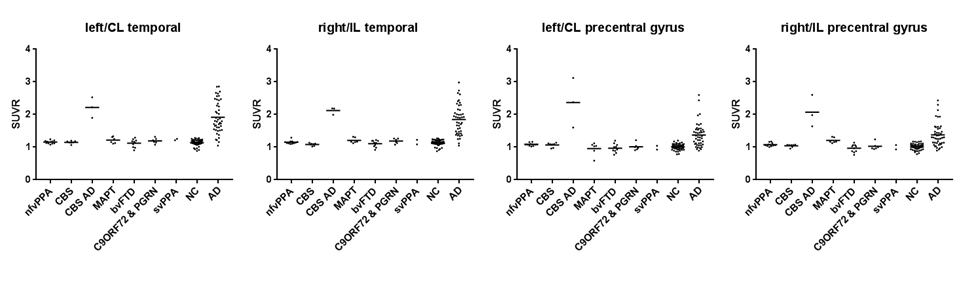

Supplement: Supplementary file 3 — Figure S3. 18F-flortaucipir in FTD, NC and AD 18F-flortaucipir SUVR in temporal and precentral gyrus across all FTD syndromes, normal controls and a cohort (n = 45) of age (median, min, max) (63, 48, 77), sex (21 female, 24 male), Mini-Mental State Examination [4, 24, 30], Clinical Dementia Rating scale sum of boxes (4, 0.5, 7) matched Alzheimer’s disease patients. For all 4 regions of interest, AD and CBS-AD group had higher SUVR compared to nfvPPA, CBS, MAPT, bvFTD, C9ORF72 & PGRN, svPPA and normal controls.(p < 0.05). No differences between AD and CBS-AD across all 4 regions of interest. CL, contralateral; IL, ipsilateral; horizontal bar denotes mean. (TIF 65 kb) [file 13195_2019_470_MOESM3_ESM.tif]
